# Research on large coal detection method for mine conveyor belt based on SCCG-YOLO

**DOI:** 10.1371/journal.pone.0330980

**Published:** 2026-04-01

**Authors:** Xinhui Zhan, Rui Yao, Yun Qi

**Affiliations:** 1 School of Coal Engineering, Shanxi Datong University, Datong, Shanxi, China; 2 School of Safety and Emergency Management, Inner Mongolia University of Science & Technology, Baotou, Inner Mongolia, China; Henan Polytechnic University, CHINA

## Abstract

To address equipment blockage and belt damage caused by large coal blocks on conveyor belts, this study proposes SCCG-YOLO, a lightweight real-time detection model based on YOLOv8n. The model introduces CPNGhost into the backbone to enhance receptive-field coverage and edge-detail extraction for large targets, incorporates Shuffle Attention in feature fusion to improve discriminability under complex lighting and dust interference, replaces fixed upsampling in the neck with CARAFE to refine high-level semantic reconstruction, and adopts DIoU loss to strengthen geometric constraints during bounding-box regression. Experiments were conducted on a task-specific derivative subset of the public CUMT-Belt dataset. After image screening, label correction, and re-annotation, 1,276 valid images were retained and divided into training, validation, and test sets at a ratio of 8:1:1. The results show that SCCG-YOLO achieves 91.9% mAP@50, 532.6 FPS, and only 2.7 MB parameters, demonstrating a favorable balance among detection accuracy, efficiency, and model compactness. These results indicate that the proposed method can satisfy the real-time detection requirements of underground conveyor-belt operation and has practical value for intelligent mine safety warning.

## 1 Introduction

With the development of intelligent mining and mechanized coal production, belt conveyors have become essential equipment for continuous coal transportation in underground and open-pit mines. During operation, large coal blocks and other impurities may enter the conveying stream, leading to belt deviation, equipment jamming, structural damage, and even shutdowns. These problems reduce production efficiency, increase maintenance cost, and threaten operational safety [1–4].

Traditional impurity detection mainly depends on manual inspection or simple alarm-based methods, which are labor-intensive and often unreliable in harsh mine environments [5]. In recent years, deep learning-based object detection, especially the YOLO series, has shown strong performance in industrial vision tasks [6–8]. Recent studies have further advanced real-time detection frameworks. YOLOv10 improved end-to-end detection efficiency through an NMS-free design [9], YOLO11 extended the engineering usability of the YOLO framework [10], and YOLOv12 introduced attention-centric design to strengthen feature modeling while maintaining real-time performance [11]. Meanwhile, Transformer-based detectors have also progressed rapidly. RT-DETRv2, RT-DETRv3, and DEIM improved the practicality of DETR-like methods through better training strategies, denser supervision, and improved matching mechanisms [12–14].

For coal-mine impurity detection, existing studies have reported encouraging results. Li Deyong et al. [15] proposed BRS-YOLOv10n for gangue recognition in underground mines. Mao et al. [16] improved YOLOv5 by combining dehazing preprocessing, CBAM, and ASFF. Yao et al. [17] developed an improved YOLOX model with image enhancement, attention mechanisms, lightweight convolution, and a rotation-decoupled head. Huang et al. [18] enhanced YOLOv5 with image enhancement and lightweight multi-scale feature extraction, and verified its effectiveness on an embedded platform. In addition, recent studies have continued to explore YOLOv10-based lightweight detection under adverse conditions such as infrared imaging, miner monitoring, low-light environments, and multispectral gangue detection [19–22].

Despite these advances, existing methods still face several limitations in mine conveyor-belt scenarios. Their robustness under low illumination, heavy dust, and blurred target boundaries remains insufficient, and research on lightweight detection of large coal blocks on conveyor belts is still limited [23,24]. Therefore, a compact and robust detector specifically designed for such environments is still needed.

To address this problem, this study proposes SCCG-YOLO, a lightweight real-time detection model based on YOLOv8n for large coal block detection on mine conveyor belts. CPNGhost is introduced into the backbone to enhance shallow feature representation, Shuffle Attention is incorporated into feature fusion to suppress background interference, CARAFE is adopted in the neck to improve feature reconstruction during upsampling, and DIoU loss is used to improve localization accuracy. Through these targeted improvements, SCCG-YOLO aims to achieve a better balance among accuracy, efficiency, and deployment suitability in complex conveyor-belt environments. In this study, “large coal” refers to coal lumps occupying a non-negligible portion of the conveyor-belt image, and the specific annotation criterion is described in Section 3.1.

## 2 Methods

### 2.1 CPNGhost module

**Fig 1:**
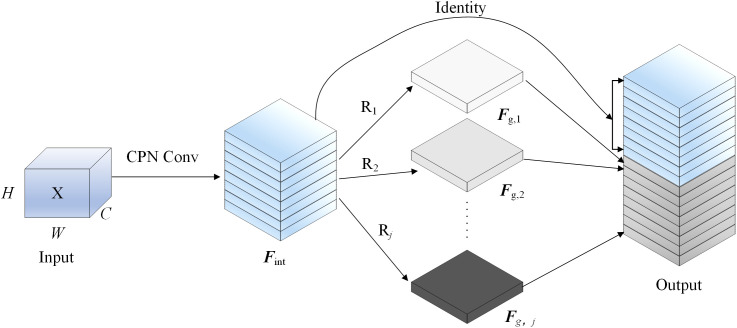
CPNGhost network architecture.

To improve the representation of large coal blocks under illumination variation and dust interference, a lightweight CPNGhost module is introduced into the YOLOv8n backbone. YOLOv8n is efficient, but its original backbone is less effective in capturing blurred boundaries, irregular shapes, and fine texture details in conveyor-belt scenes. The proposed module combines progressive normalization and Ghost convolution, which stabilizes shallow features and enriches channel representations with low computational cost. As a result, the backbone obtains stronger edge and texture modeling ability while maintaining lightweight characteristics [25–28]. See S1 File for specific steps. The structure of CPNGhost is shown in Fig 1.

### 2.2 SA module

**Fig 2:**
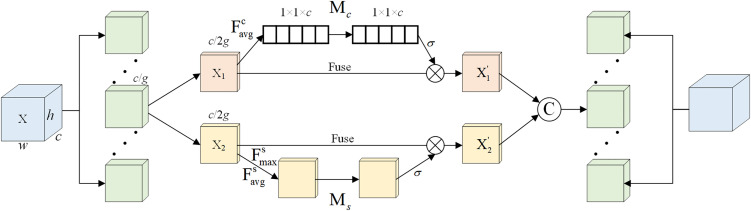
SA network architecture.

In mine conveyor-belt environments, low illumination, dust, and complex backgrounds often reduce detection accuracy. To improve feature discrimination, Shuffle Attention (SA) is introduced into the feature-fusion stage of YOLOv8n. The SA module jointly models channel and spatial attention, enabling the network to focus more effectively on salient impurity regions while suppressing background interference. Because of its lightweight design, SA is well suited to real-time industrial deployment [29–32]. See S2 File for specific steps. The structure of the SA module is shown in Fig 2.

### 2.3 CARAFE dynamic upsampling

**Fig 3:**
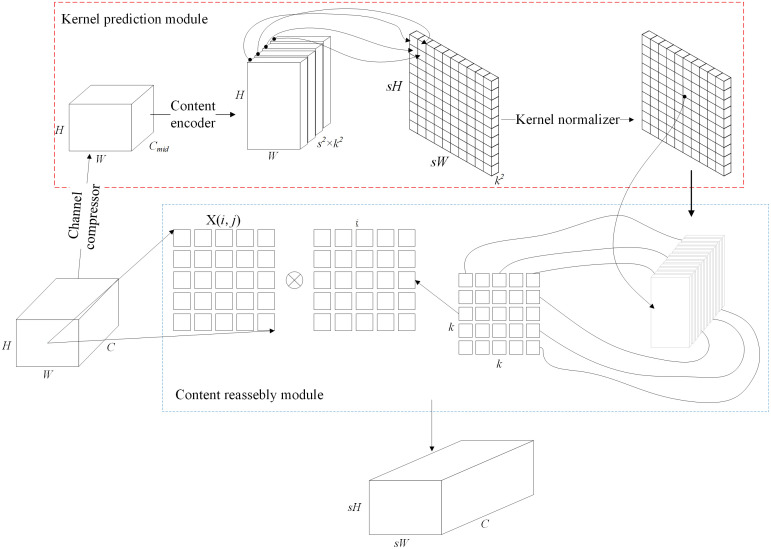
CARAFE network architecture.

The original YOLOv8n uses fixed upsampling methods, such as nearest-neighbor or bilinear interpolation, which may blur details during feature reconstruction. To address this limitation, CARAFE is adopted to replace fixed upsampling in the neck. CARAFE generates content-aware kernels to reassemble features adaptively, which helps preserve the contours and textures of large coal blocks during multi-scale fusion. This improves semantic reconstruction and target localization with only a small computational increase [33–36]. See S3 File for specific steps. The structure of CARAFE is shown in Fig 3.

### 2.4 DIoU loss

The original YOLOv8n uses GIoU loss for bounding-box regression, but its sensitivity to center-point deviation is limited, which may slow convergence and reduce localization accuracy in complex scenes. Therefore, DIoU loss is introduced to replace GIoU. By incorporating the distance between the centers of the predicted box and the ground-truth box, DIoU provides more effective geometric constraints, especially when the boxes do not overlap. This improves regression stability, accelerates convergence, and enhances localization accuracy for large coal blocks in cluttered conveyor-belt environments [37,38]. See S4 File for specific steps.

### 2.5 Improved model

To address the challenges in recognizing large coal block impurities on coal mine conveyor belts caused by varying impurity sizes, blurred object edges, and strong background noise interference during operation, this study proposes an improved YOLOv8n-based object detection method named SCCG-YOLO (Shuffle Attention and CARAFE with CPNGhost-YOLO). Based on the lightweight YOLOv8n architecture, SCCG-YOLO introduces critical optimizations in both network structure and loss function design:

Replace the standard Conv-BN-SiLU units inside the backbone C2f blocks with CPNGhost at all downsampling stages that feed P2, P3, P4, P5 (strides 4/8/16/32). This preserves tensor shapes and strides while stabilizing shallow features under illumination/dust and expanding channel diversity at low cost. The outputs at P3/8, P4/16, P5/32 continue to serve as inputs to the neck exactly as in YOLOv8n; immediately after the last CPNGhost stage at P5/32 and before SPPF, insert ShuffleAttention. Grouped channel–spatial re-weighting with inter-group shuffling suppresses background clutter and amplifies salient coal/impurity structures, leveraging the diversified channels produced by CPNGhost; replace the two stride-2 fixed upsampling ops in the PAN/FPN neck with CARAFE, upsamples the P5/32 feature to P4/16, followed by Concat with the backbone P4 feature and a C2f refinement and the fused P4/16 feature to P3/8, followed by Concat with the backbone P3 feature and a C2f refinement; replace GIoU with DIoU for bounding-box regression on all three scales (P3/P4/P5), adding a center-distance term that is consistent with the sharper contours produced upstream. Classification/objectness branches remain unchanged; this affects training loss only and does not alter inference topology.

**Fig 4:**
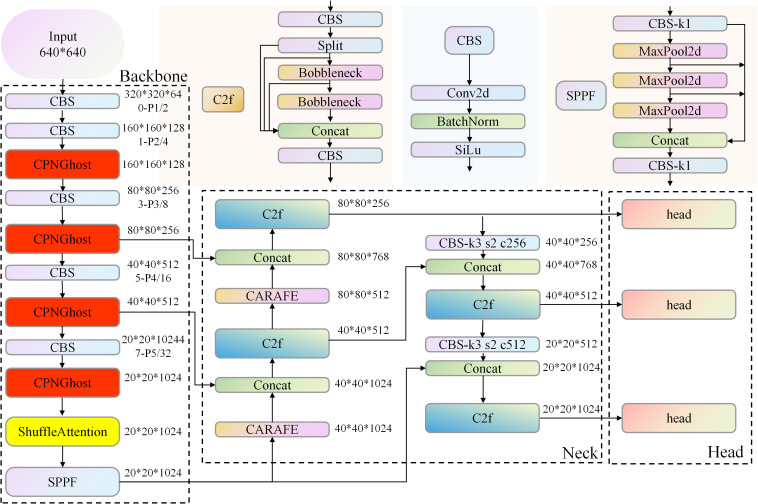
SCCG-YOLO network architecture.

Firstly, CPNGhost combines progressive normalization with Ghost convolution to stabilize shallow representations and expand channel diversity at low cost, enlarging the receptive field and improving boundary modeling for large coal blocks. These richer yet stable features are a prerequisite for effective grouped re-weighting in the neck [39].Secondly, ShuffleAttention performs grouped channel–spatial attention followed by inter-group shuffling, suppressing redundant responses while amplifying salient impurity regions in complex backgrounds (scratches, slurry). It explicitly leverages the channel diversity produced by CPNGhost, turning it into discriminative focus [40].Thirdly, replacing fixed upsampling with CARAFE allows dynamic, content-aware kernels to reassemble high-resolution features, preserving edges and contours that were emphasized by attention, and improving target localization and recall. [41].Finally, DIoU adds a center-distance term that complements the sharpened contours and spatially consistent features produced by the above stages, accelerating convergence and reducing close-range false positives [42]. This design is not a mere aggregation of modules. Instead, each component is positioned to address a concrete bottleneck observed in the dataset and the conveyor-belt scenario, and their interactions are intentionally staged along the backbone–neck–upsampling–regression pipeline. The improved model architecture is illustrated in Fig 4.

### 2.6 Conceptual comparison with recent YOLO variants

SCCG-YOLO differs from YOLOv10, YOLOv12, and YOLO-NAS in design philosophy. The latter are general-purpose detectors that emphasize unified architecture design, global optimization, or neural architecture search, whereas SCCG-YOLO adopts a scene-oriented optimization strategy for coal conveyor-belt environments, where large targets often exhibit blurred boundaries, illumination variation, and severe dust interference [43].

At the backbone level, YOLOv10, YOLOv12, and YOLO-NAS mainly rely on standard convolutional structures or architecture search to ensure general feature extraction ability. In contrast, SCCG-YOLO introduces CPNGhost to enhance edge, texture, and contour representation of large coal targets through progressive normalization and Ghost feature generation, making the backbone more suitable for this task [44]. At the neck level, YOLOv10 and YOLOv12 mainly improve multi-scale fusion efficiency, while YOLO-NAS emphasizes broad adaptability through searched structures. SCCG-YOLO instead employs CARAFE for content-aware upsampling, which better preserves structural details of large coal blocks under weak illumination and dusty backgrounds [45]. In terms of attention, YOLOv10 and YOLOv12 do not explicitly integrate lightweight attention modules, and YOLO-NAS remains largely task-agnostic. SCCG-YOLO incorporates ShuffleAttention to strengthen salient responses and suppress background disturbance through channel grouping, spatial weighting, and channel shuffling, which is particularly beneficial in complex coal-mine scenes [46].

Overall, compared with recent general-purpose YOLO variants, SCCG-YOLO follows a task-driven module customization strategy. Its backbone, neck, and attention design are jointly optimized for conveyor-belt large coal detection, providing stronger robustness and better engineering adaptability in challenging mine environments.

## 3 Experimental preparation and parameter settings

### 3.1 Dataset construction

The dataset used in this study is a task-specific derivative subset constructed from the publicly available CUMT-Belt dataset released by China University of Mining and Technology. The original images were collected by overhead cameras during conveyor-belt transportation. For the large-coal detection task, images with severe blur, incomplete target visibility, excessive occlusion, or obviously inconsistent labels were removed. The remaining images were manually re-annotated in YOLO format using LabelImg to ensure annotation consistency under the target definition adopted in this work. After screening and re-annotation, 1,276 valid images were retained.

**Fig 5:**
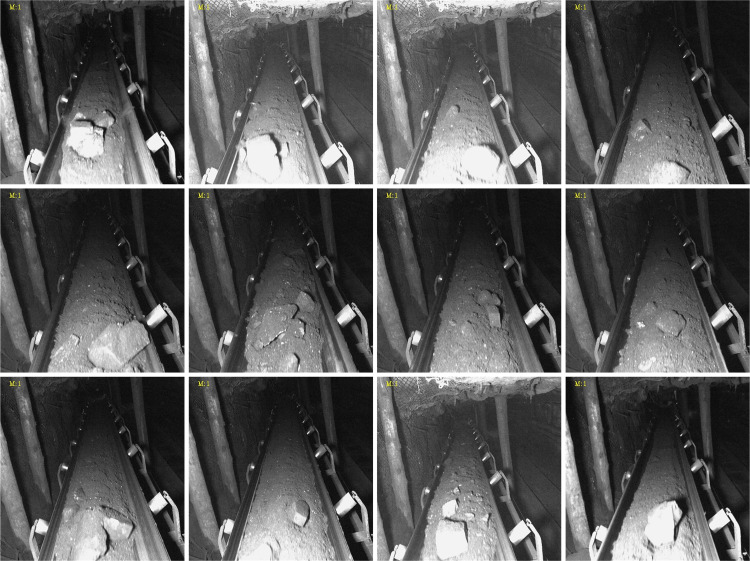
Partial dataset.

These images were divided into training, validation, and test sets at a ratio of 8:1:1 (1,020/128/128). The contribution of this study lies in the task-oriented screening protocol, the derivative annotation set, and the fixed data split, while the raw images remain from the public dataset. To facilitate reproducibility, the derivative annotation files, image list, split indices, and training configuration will be released together with the public-image references upon acceptance. A subset of the curated dataset is shown in Fig 5.

### 3.2 Overfitting risk and generalization control

Because the curated dataset is limited in scale, overfitting remains a potential concern. To mitigate this risk, data augmentation, L2 weight decay, cosine annealing, and early stopping were applied during training. In addition, the lightweight design of SCCG-YOLO reduces parameter redundancy under small-sample conditions.

To further assess generalization, the study reports training/validation mAP@50 curves, precision–recall curves, confusion matrices, and scatter plots of target size and spatial position. Under the current training configuration, no sustained train–validation divergence was observed. This suggests that the performance gain mainly arises from more effective feature representation rather than simple memorization.

### 3.3 Parameter settings

All experiments were conducted using PyTorch 2.0 with Python 3.8 and CUDA 11.8 on Windows 11. The hardware platform consisted of an Intel Xeon Gold 6430 CPU, 32 GB RAM, and an NVIDIA GeForce RTX 4090 GPU with 24 GB memory. To ensure a fair comparison, all baseline and comparison models were trained and evaluated under the same software and hardware conditions, dataset split, image resolution, optimizer, batch size, learning-rate schedule, early-stopping criterion, and maximum number of training epochs as SCCG-YOLO. No model-specific acceleration, pruning, quantization, or TensorRT-based optimization was applied unless otherwise stated. The detailed hyperparameter settings are listed in Table 1.

**Table 1:** Hyperparameter setting.

Parameter	Values
Images Size	640 × 640 × 3
Lr	0.01
Momentun	0.937
Batch size	32
Epoch	150
Weight decay	0.005

### 3.3 Evaluation metrics

Five commonly used performance metrics in the field of object detection were selected to comprehensively evaluate the model’s detection capability, real-time performance, and deployment feasibility. These metrics include Precision, Recall, the Number of Parameters (Parameters), Mean Average Precision (mAP), and Frames Per Second (FPS) [47]. The corresponding formulas are presented as follows:


$$\text{Precision}=\frac{TP}{TP+FP}\;$$ (1)

$$\text{Recall}=\frac{TP}{TP+FN}\;$$ (2)

$$\text{mAP}=\frac{\text{1}}{N}\sum_{\;i=1}^{N}AP_{i}\;$$ (3)

In the formulas, *TP* denotes the number of correctly detected targets; *FP* represents the number of false positives; FN indicates the number of missed detections; *N* is the total number of categories; and *AP*_*i*_ refers to the Average Precision of the *i*-th category.

## 4 Results and discussion

### 4.1 Training results of SCCG-YOLO

**Fig 6:**
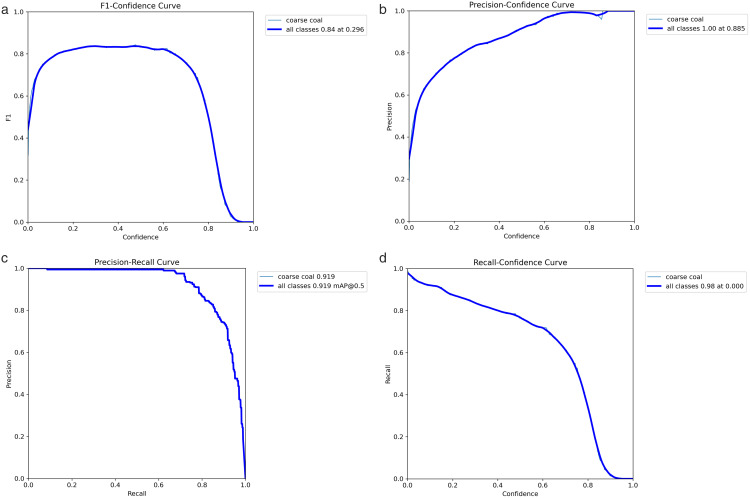
Training results of SCCG-YOLO. (a) F1-Confidence curve; (b) Precision-Confidence curve; (c) Precision-Recall curve; (d) Recall-Confidence curve.

To comprehensively evaluate the recognition stability and threshold sensitivity of the SCCG-YOLO model in the task of detecting large coal foreign objects on conveyor belts, the F1-Confidence curve, Precision-Confidence curve, Precision-Recall curve, and Recall-Confidence curve were output based on the test set, and a systematic analysis of the model’s comprehensive performance under different confidence thresholds was carried out. The test results are shown in Fig 6.

The F1–Confidence curve shows that the model achieves its highest F1 score of 0.84 at a confidence threshold of approximately 0.296, and maintains a consistently high level within the range of 0.3–0.6, indicating that SCCG-YOLO preserves a well-balanced trade-off between precision and recall across a broad threshold interval. The Precision–Confidence curve demonstrates that precision increases steadily with the confidence threshold, reaching 1.00 at 0.885, suggesting that the model produces nearly no false positives under high-threshold conditions and is therefore suitable for real-time early-warning applications in practical industrial scenarios. The Precision–Recall curve remains smooth overall, with mAP@0.5 reaching 0.919, revealing that the model maintains high precision even in high-recall regions. This verifies its robustness against illumination disturbances, dust-laden backgrounds, and irregular-shaped large coal blocks. Moreover, the Recall–Confidence curve indicates that the model attains a recall as high as 0.98 at low thresholds, followed by a gradual decline as the threshold increases, reflecting its strong capability in capturing potential large-coal foreign objects. Taken together, the four types of curves show that SCCG-YOLO achieves a well-balanced performance among precision, recall, and threshold sensitivity. It maintains extremely high recall at low thresholds while ensuring minimal false detections at high thresholds. These findings are consistent with the aforementioned mAP results and fully demonstrate the effectiveness of the proposed improvements in enhancing detection stability, feature representation capacity, and adaptability to complex environments.

### 4.2 Dataset characteristics analysis

**Fig 7:**
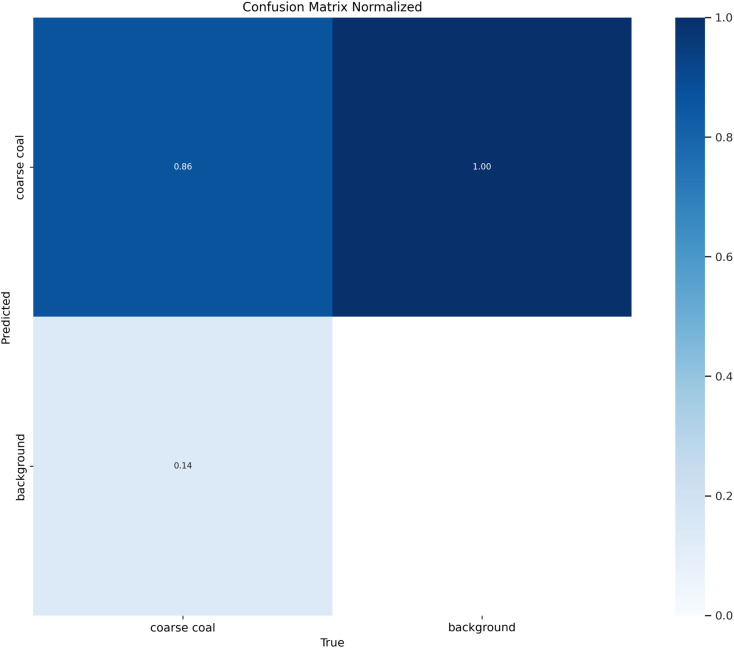
Confusion matrix normalized.

**Fig 8:**
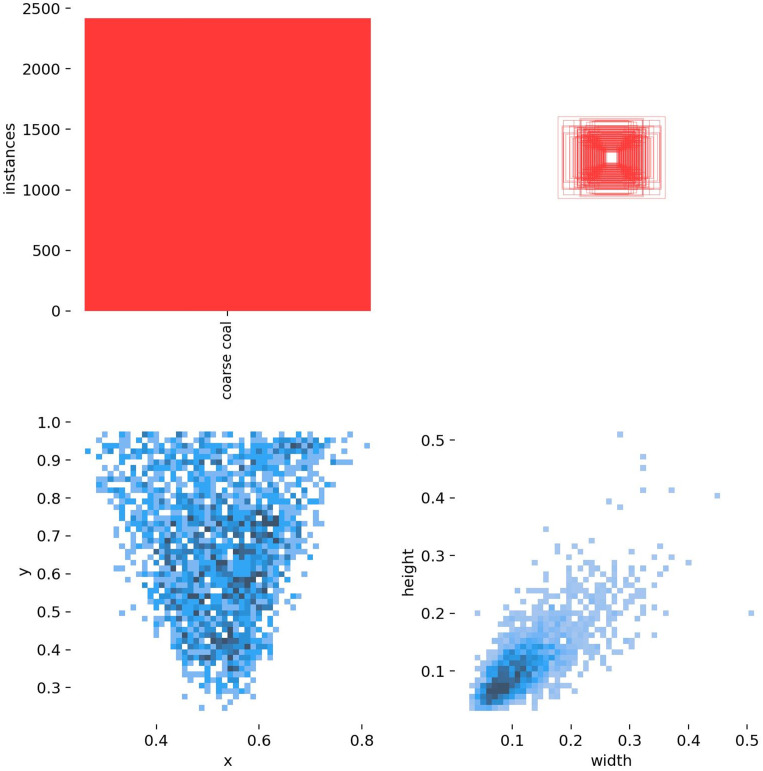
Label distribution plot.

**Fig 9:**
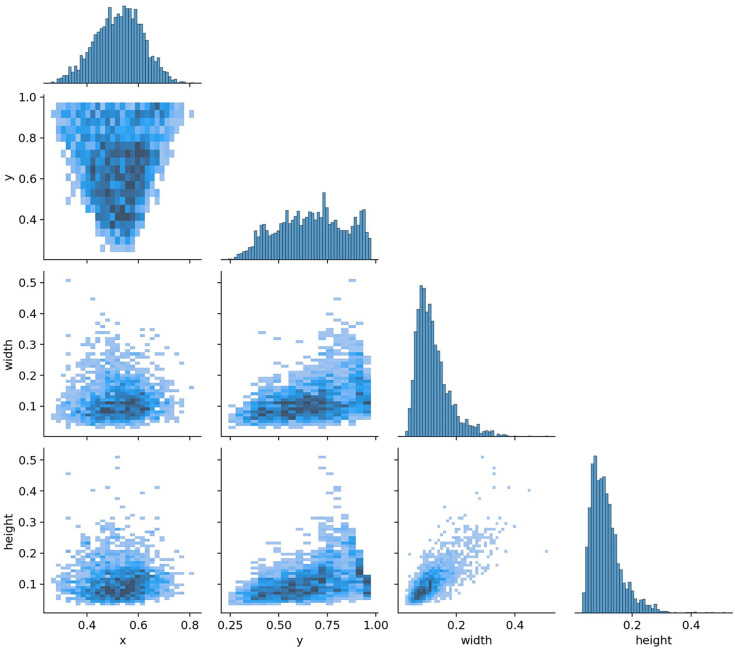
Label scatterplot matrix.

To evaluate the adaptability of the SCCG YOLO model for detecting coarse coal foreign objects on coal mine conveyor belts, an in-depth analysis of the training dataset was conducted, focusing on class distribution, spatial distribution of bounding boxes, and target size characteristics. The normalized confusion matrix (Fig 7), label distribution characteristics (Fig 8), and label scatter plot matrix (Fig 9) illustrate these aspects.

As shown in Fig 7, the positive class ‘coarse coal’ overwhelmingly dominates the dataset. The model achieves a recall rate of 86% for coarse coal, while the background class exhibits a recall rate of 14%, indicating that the positive samples provide rich feature information that effectively guides parameter learning during training. The abundance of positive instances serves as the primary driving force for training, enabling the model to capture diverse morphological features of coal blocks from various angles.

Fig 8 reveals a concentrated spatial distribution of bounding boxes. As can be seen from the upper left of Fig 7, the positive class coarse coal comprises 2300 annotated instances in the training split.The normalized horizontal coordinate (x) of the object center is primarily distributed between 0.35 and 0.75, while the vertical coordinate (y) falls within the range of 0.35 to 0.90, as illustrated in the lower left of Fig 8. This distribution pattern aligns with the overhead camera perspective, where coal blocks enter from the edges of the frame and gradually converge toward the upper center region, providing the model with stable prior knowledge for feature extraction in key areas.

Furthermore, the normalized object width is predominantly within 0.05–0.20, and the height ranges between 0.05–0.15, as shown in the lower right of Fig 8. This indicates that the dataset comprises a diverse array of coal block shapes with relatively consistent sizes, including both single large blocks and common forms with an aspect ratio between 1:1 and 1.5. The observed spatial clustering underscores the efficacy of the SA module in focusing on upper-center regions, as integrated in Section 2.5, while the size correlations inform the threshold in 3.1 to prioritize non-negligible lumps.

Fig 9 demonstrates strong correlations among various features. Width and height show a clear positive correlation, reflecting the stable morphological proportions of coal blocks under different perspectives. The vertical position (y) is mildly positively correlated with size, suggesting that coal blocks located in the upper part of the image tend to be slightly larger, which is consistent with the spatial relationship between the camera and the target. In contrast, the horizontal position (x) exhibits weak correlation with width and height, indicating minimal scale variation of coal blocks along the horizontal axis.

### 4.3 Comparison experiments of improved modules

#### 4.3.1. Backbone.

To validate the effectiveness of the proposed improvement strategies in the backbone network, a series of comparative experiments were conducted by integrating commonly used modules into the backbone. The experimental results are summarized in Table 2.

**Table 2:** Comparison experiment of backbone network modules.

Model	P/%	R/%	mAP@50/%	mAP@50:90/%	FPS	Parama/MB	F1-Score/%	GFLOPs
YOLOv8n	86.8	78.9	90.3	59.0	533.3	3	82.7	8.1
+ MobileNetv4	87.9	78.5	89.6	58.2	508.4	2.8	82.9	7.7
+ FasterNeXt	87.8	80.7	91.6	59.6	510.7	2.6	84.1	6.8
+ ConvNeXtv2	89.6	78.1	90.4	59.7	335.3	2.7	83.5	7.3
+ C3_Ghost	88.0	78.1	90.2	57.4	548.0	2.5	82.8	6.6
+ CSCGhost	93.3	76.3	91.4	58.9	504.9	2.5	83.9	6.7
+ CPNGhost	87.1	82.6	91.9	58.5	521.2	2.4	84.8	6.6

The baseline YOLOv8n model achieved a precision of 86.8%, a recall of 78.9%, and an mAP@50 of 90.3%. After incorporating the CPNGhost module, the recall significantly increased to 82.6%, and the mAP@50 improved to 91.9%. Although the precision slightly decreased to 87.1%, the overall detection performance became more balanced. This indicates that the CPNGhost module effectively enhances the backbone’s receptive field and its capability to capture edge features of targets.

**Fig 10:**
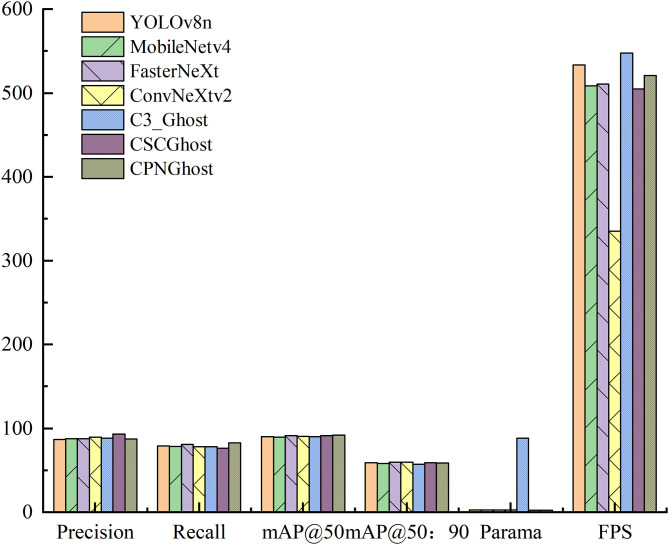
Comparison experiment of backbone network modules.

Moreover, the CPNGhost module demonstrates a remarkable advantage in model complexity, with parameters reduced to only 2.4 MB, making it the most lightweight among all the improved models. It also achieved an inference speed of 521.2 FPS, exhibiting excellent real-time performance. Compared with other classic modules such as CSCGhost, ConvNeXtv2, and MobileNetv4, CPNGhost strikes a favorable balance between inference efficiency and recall improvement, particularly enhancing the detection of foreign objects in complex environments. The experimental results are further illustrated in Fig 10, which presents a bar chart comparison of the performance across different backbone modules.

#### 4.3.2. Attention mechanism.

To further verify the enhancement effect of the SA (ShuffleAttention) attention mechanism module on the detection performance of coarse coal foreign objects in coal mines, a comparative analysis was conducted involving several mainstream attention mechanisms, including GAM (Global Attention Mechanism), MLLA (Mamba-Like Linear Attention), and SimAM. The experimental results are detailed in Table 3.

**Table 3:** Comparison experiment of attention mechanism modules.

Model	P/%	R/%	mAP@50/%	mAP@50:90/%	FPS	Parama/MB	F1-Score/%	GFLOPs
YOLOv8n	86.8	78.9	90.3	59.0	533.3	3.0	82.7	8.1
+ GAM	83.9	83.2	91.1	59.7	544.3	3.4	83.5	8.4
+ MLLA	81.0	85.1	91.0	59.3	538.8	3.1	83.0	8.2
+ SimAM	85.4	82.3	91.4	58.6	592.6	3.0	83.8	8.1
+ SA	89.5	81.9	92.5	60.0	517.2	3.0	85.5	8.1

The findings demonstrate that the introduction of the ShuffleAttention mechanism significantly improved the mAP@50 to 92.5%, outperforming other attention mechanisms in overall performance. In addition, the SA module achieved a precision of 89.5% and effectively elevated the mAP@50:90 metric to 60%, highlighting its capability to focus on foreign object target regions while suppressing background noise.

**Fig 11:**
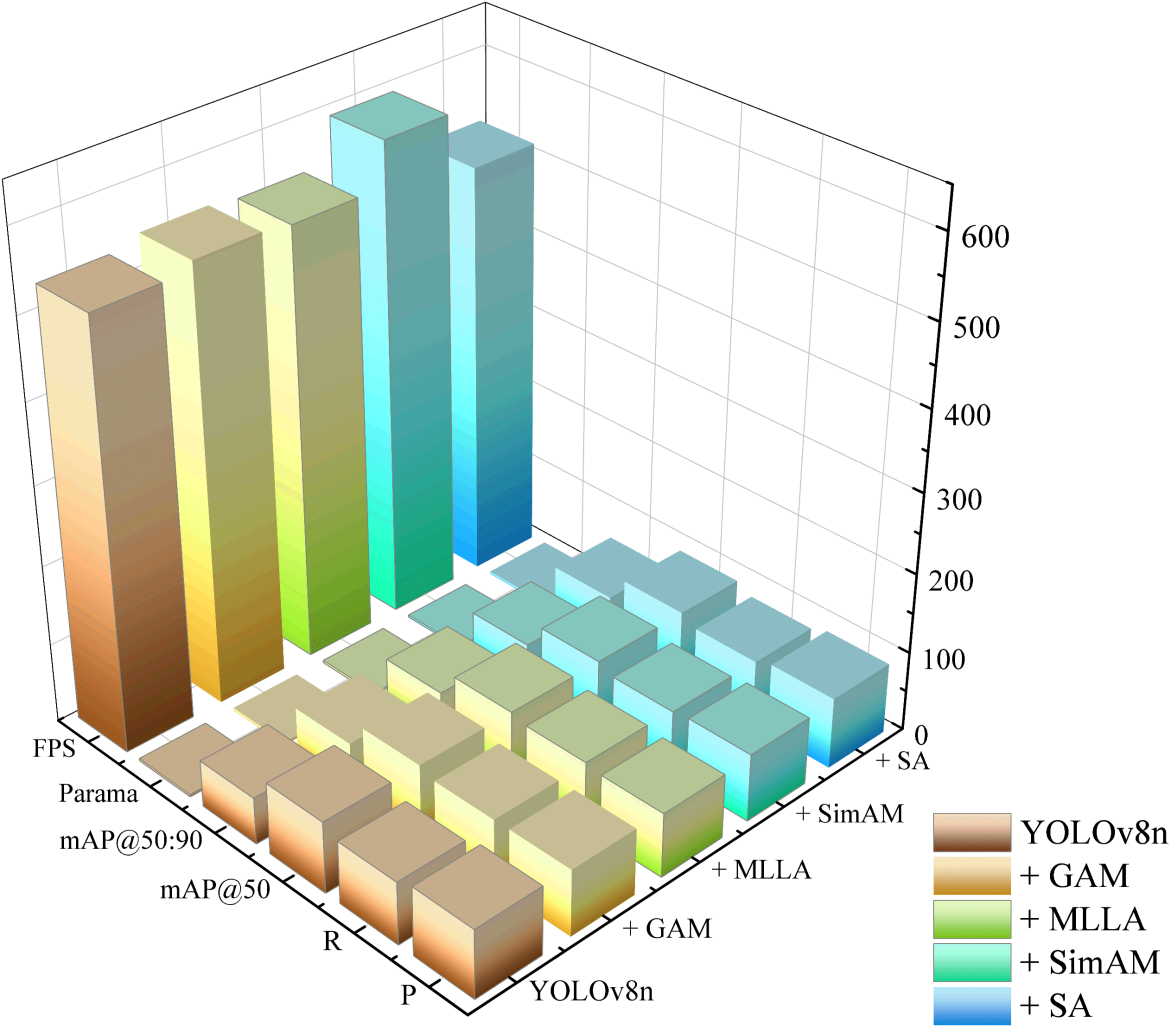
Comparison experiment of attention mechanism modules.

Although the inference speed slightly decreased compared to the baseline model after integrating the SA module, the model parameter size remained lightweight at 3.0 MB. This ensures that the model maintains its applicability for real-time detection in practical industrial scenarios. The comparison results of the attention mechanism experiments are visualized in Fig 11, which presents a 3D comparison chart illustrating the performance differences among the tested attention modules.

#### 4.3.3. Upsampling.

To evaluate the enhancement effect of dynamic upsampling operators on the detection of coarse coal foreign objects in coal mines, the performance impact of two dynamic upsampling methods—Dysample and CARAFE—on the YOLOv8n baseline model was compared. The experimental results are summarized in Table 4.

**Table 4:** Comparison experiment of dynamic upsampling operators.

Model	P/%	R/%	mAP@50/%	mAP@50:90/%	FPS/	Parama/MB	F1-Score/%	GFLOPs
YOLOv8n	86.8	78.9	90.3	59.0	533.3	3.0	82.7	8.1
+ Dysample	88.8	79.1	90.2	59.8	500.1	3.0	83.7	8.1
+ CARAFE	86.7	82.2	91.1	59.6	474.5	3.1	84.4	8.6

When applying the Dysample dynamic upsampling operation, the model’s precision improved to 88.8%; however, the mAP@50 slightly decreased to 90.2%, indicating that Dysample provided limited gains in detection accuracy. Additionally, recall did not exhibit significant improvement, and the inference speed decreased to 500.1 FPS, with no change in parameter size.

In contrast, the integration of the CARAFE dynamic upsampling operator led to notable performance enhancements. The recall rate significantly increased to 82.2%, and the mAP@50 reached 91.1%, outperforming both the baseline model and the Dysample-enhanced model. This demonstrates that CARAFE effectively enhances the model’s ability to preserve high-resolution semantic features and improves the accuracy of target localization. Although the inference speed of the CARAFE model was moderately reduced, the parameter size only increased to 3.1 MB, resulting in minimal impact on the model’s practical real-time detection performance.

#### 4.3.4. Loss function.

To assess the impact of different IoU-based loss functions on the detection performance of coarse coal foreign objects in coal mines, comparative experiments were conducted on the baseline YOLOv8n model using CIoU, MPDIoU, SDIoU, and DIoU loss functions. The experimental results are detailed in Table 5.

**Table 5:** Comparison experiment of loss functions.

Model	P/%	R/%	mAP@50/%	mAP@50:90/%	FPS	Parama/MB	F1-Score/%	GFLOPs
YOLOv8n	86.8	78.9	90.3	59.0	533.3	3.0	82.7	8.1
+ CIoU	86.8	78.9	90.3	59.0	559.3	3.0	82.7	8.1
+ MPDIoU	83.2	84.1	91.1	60.2	581.2	3.0	83.6	8.1
+ SDIoU	86.4	82.7	91.3	60.3	503.1	3.0	84.5	8.1
+ DIoU	82.6	84.4	91.6	59.7	492.9	3.0	83.5	8.1

The baseline model achieved an mAP@50 of 90.3%. Incorporating the CIoU loss function did not yield significant changes, with precision and recall remaining at 86.8% and 78.9%, respectively. In contrast, the MPDIoU loss function notably improved the recall rate to 84.1%, and the mAP@50 increased to 91.1%, surpassing the baseline performance.

The SDIoU loss function further elevated the mAP@50 to 91.3%, with the mAP@50:90 reaching the highest value of 60.3%. Although the recall rate of 82.7% was slightly lower than that of MPDIoU, the overall detection performance remained outstanding. The DIoU loss function delivered the highest recall rate and mAP@50 among all tested configurations, effectively enhancing the model’s localization precision for foreign object targets.

**Fig 12:**
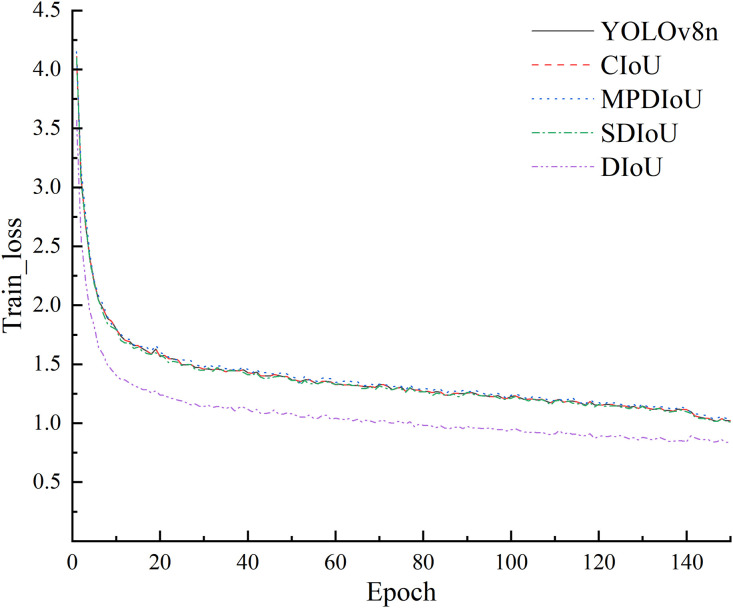
Comparison experiment of loss functions.

While the introduction of the DIoU loss function resulted in a slight reduction of inference speed to 492.9 FPS, the parameter size remained unchanged at 3.0 MB, ensuring the model’s suitability for real-time detection applications. The training process of different models with respective loss functions is illustrated in Fig 12.

In summary, the DIoU loss function significantly improves detection precision and recall for coarse coal foreign objects in complex coal mine environments, making it the preferred choice for loss function optimization in this application context.

### 4.4 Ablation study

To validate the actual contributions of each improved module within the enhanced model for detecting coarse coal foreign objects on coal mine conveyor belts, a comprehensive ablation study was conducted. By incrementally integrating each key module and comparing the detection performance of different model variants under identical datasets and training strategies, the impact of each sub-module on detection accuracy, model complexity, and inference speed was systematically evaluated, thereby confirming the effectiveness of SCCG-YOLO.

All models were trained for 150 epochs on the dataset using consistent training hyperparameters and evaluation metrics, including Precision, Recall, mAP@50, mAP@50:95, parameter count, and FPS, to ensure fairness and reliability of the comparison. The ablation experiment results are summarized in Table 6.

**Table 6:** Ablation experiment results.

Model	P/%	R/%	mAP@50/%	mAP@50:90/%	FPS	Parama/MB	F1-Score/%	GFLOPs
YOLOv8n	86.8	78.9	90.3	59.0	533.3	3.0	82.7	8.1
+ CPNGhost	87.1	82.6	91.9	58.5	521.2	2.4	84.8	6.6
+ SA	89.5	81.9	92.5	60.0	517.2	3.0	85.5	8.1
+ CARAFE	86.7	82.2	91.1	59.6	474.5	3.1	84.4	8.6
+ DIoU	82.6	84.4	91.6	59.7	492.9	3.0	83.5	8.1
+ CPNGhost+SA	87.8	79.7	90.1	59.1	296.1	2.5	83.6	6.7
+ CPNGhost+SA+CARAFE	90.4	79.3	92	59.2	284	2.7	84.5	7.2
SCCG-YOLO	83.3	84.1	91.9	59.1	532.6	2.7	83.7	7.2

As shown in Table 6, the CPNGhost module significantly improved the recall from 78.9% (baseline YOLOv8n) to 82.6%, with an mAP@50 increase of 1.6 percentage points to 91.9%. Meanwhile, the parameter count was reduced from 3.0 MB to 2.4 MB, demonstrating that CPNGhost not only enhances performance but also offers a lightweight advantage.

The SA module exhibited the most remarkable standalone performance, elevating precision to 89.5% and mAP@50 to 92.5%, making it the most effective in terms of comprehensive performance enhancement. While the CARAFE dynamic upsampling operator alone provided moderate improvements, its combination with CPNGhost and SA modules led to a significant precision boost to 90.4%, further confirming its strength in preserving high-resolution semantic features.

Introducing the DIoU loss function raised the recall rate to 84.1%. However, the overall precision slightly decreased to 83.3%, and mAP@50 saw a minor reduction. Notably, the model’s inference speed increased to 532.6 FPS, markedly enhancing the inference efficiency. It is worth noting that the naïve combination CPNGhost+SA in Table 6 yields an mAP@50 (90.1%) slightly lower than using CPNGhost or SA alone. This does not indicate a failure of the two modules to cooperate, but rather reflects a changed precision–recall balance when SA operates on already sharpened backbone features: the joint variant becomes more conservative, filtering out low-confidence detections near object boundaries and slightly reducing recall under the fixed training and inference settings used for all ablations. The intermediate combination model (CPNGhost+SA+CARAFE) shows a substantially lower FPS than the final SCCG-YOLO, despite their similar design complexity. This difference was measured under the same hardware environment and the same inference pipeline, without additional deployment-side acceleration. The result indicates that SCCG-YOLO is not a simple superposition of modules. Instead, after jointly integrating CPNGhost, SA, CARAFE, and DIoU, the final architecture benefits from a more coordinated feature flow and more appropriate channel allocation. As a consequence, the number of redundant candidate boxes before NMS is reduced, which lowers post-processing overhead and improves end-to-end inference efficiency. Therefore, SCCG-YOLO achieves a markedly higher FPS than the intermediate combination model under identical test conditions.

**Fig 13:**
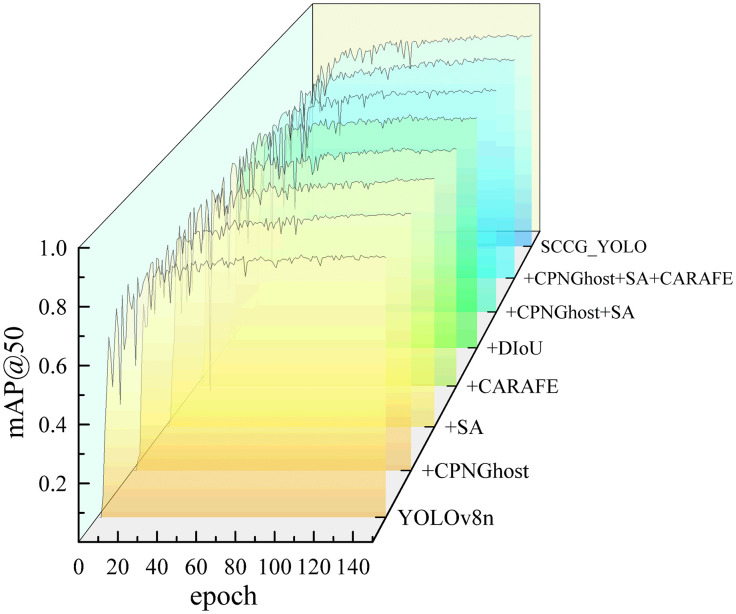
Comparison of ablation experiment results on mAP@50.

In the full SCCG-YOLO architecture, where CPNGhost and SA are further complemented by CARAFE and DIoU and trained as a whole, this effect is mitigated and the detector achieves a more favorable trade-off between detection accuracy, recall and real-time performance. Compared with the intermediate combination, SCCG-YOLO reduces the redundancy of NMS candidates through the adjustment of module positions and channel ratios, thus bringing about a decrease in postprocess. Overall, SCCG-YOLO achieves a well-balanced trade-off between localization accuracy, recall capability, and inference efficiency, making it highly suitable for real-world industrial applications in detecting coarse coal foreign objects on conveyor belts. The comparative mAP@50 performance of each model variant is illustrated in Fig 13.

### 4.5 Comparative experiments

To systematically evaluate the overall performance of SCCG-YOLO in detecting coarse coal foreign objects on coal mine conveyor belts, comparative experiments were conducted against mainstream lightweight YOLO series models. The experimental results are presented in Table 7.

**Table 7:** Comparative experiment results.

Model	P/%	R/%	mAP@50/%	mAP@50:90/%	FPS	Parama/MB	F1-Score/%	GFLOPs
YOLOv8n	86.8	78.9	90.3	59.0	533.3	3.0	82.7	8.1
YOLOv5n	86.5	81.9	91.7	58.0	258.2	1.8	84.1	4.1
YOLOv7-tiny	79.7	85.9	89.3	56.0	35.0	6.0	82.7	13.2
YOLOv9t	86.2	82.2	91.1	60.1	76.3	2.6	84.2	11.0
YOLOv10n	83.7	80.4	88.5	57.8	714.3	2.3	82.0	8.4
YOLOv11n	89.4	78.1	91.6	59.5	87.7	2.6	83.4	6.4
YOLOv12n	88.2	80.0	90.9	59.3	29.6	2.5	83.9	5.8
SCCG-YOLO	83.3	84.1	91.9	59.1	532.6	2.7	83.7	7.2

**Note:** All models in Table 7 were trained from the same train/validation/test split under identical training settings and tested on the same hardware platform without additional inference optimization.

As shown in Table 7, SCCG-YOLO achieved the highest mAP@50 of 91.9% among all models, while maintaining a real-time inference speed of 532.6 FPS. Its mAP@50:90 reached 59.1%, which is only 1 percentage point lower than that of YOLOv9t, yet SCCG-YOLO’s inference speed is approximately 7 times faster than YOLOv9t. The precision of SCCG-YOLO is 83.3%, slightly lower than that of YOLOv11n and YOLOv8n. However, its recall rate of 84.1% surpasses all models except YOLOv7-tiny, indicating stronger tolerance to missed detections and superior capability in identifying foreign objects.

Although YOLOv7-tiny achieves the highest recall rate, its inference speed is limited to 35 FPS, with a parameter size of 6.0 MB, which is inadequate for on-site online detection that demands both high throughput and low latency. While YOLOv10n attains an inference speed of 714.3 FPS, its mAP@50 is only 88.5%, reflecting insufficient detection precision.

**Fig 14:**
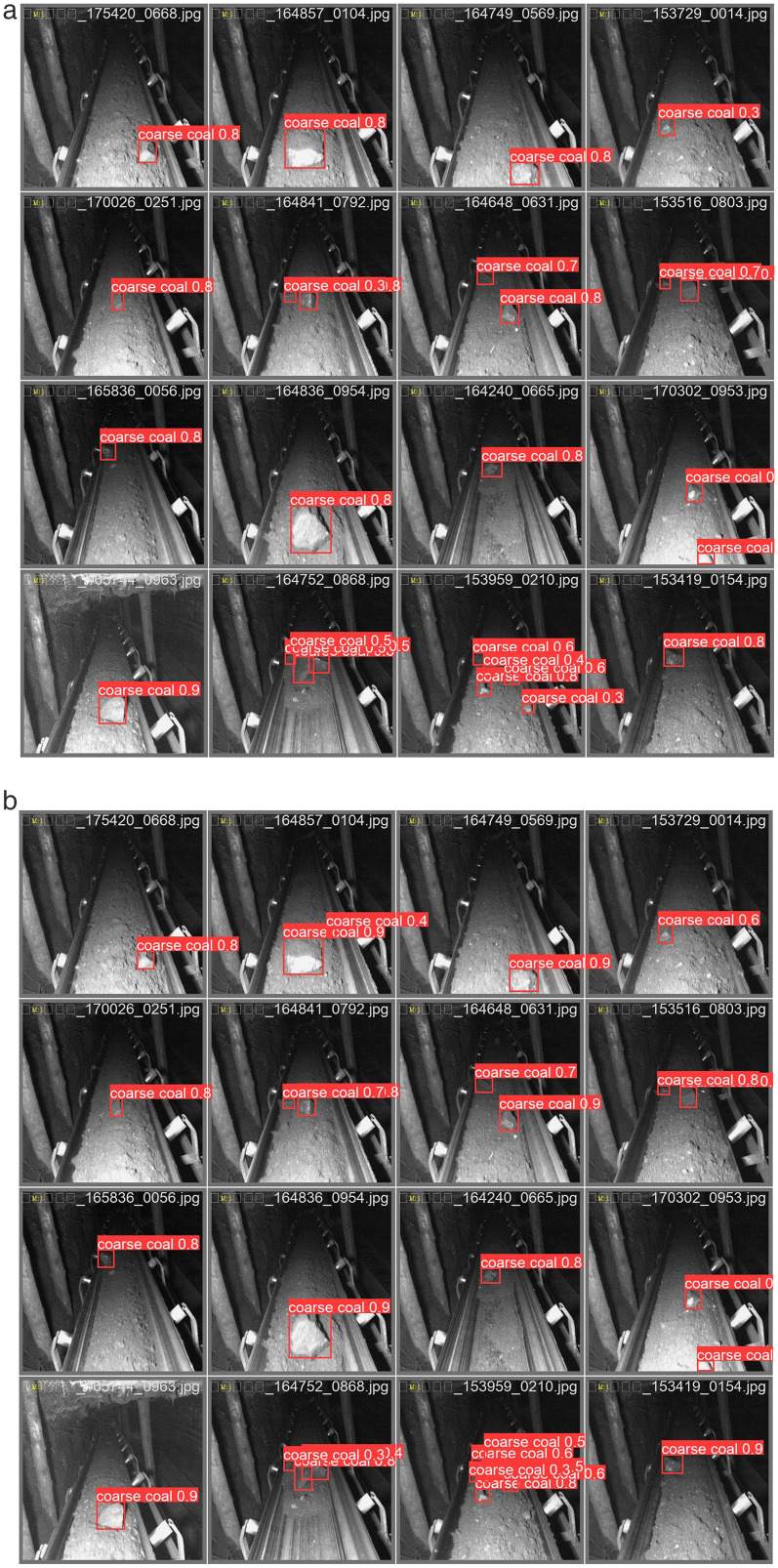
Comparison of detection effects between SCCG-YOLO and YOLOv8n. (a) Detection erformance of YOLOv8n on coarse coal foreign object detection in coal mine conveyor belts; (b) Detection erformance of SCCG-YOLO on coarse coal foreign object detection in coal mine conveyor belts.

Considering model size, detection accuracy, and inference efficiency, SCCG-YOLO achieves the optimal balance between precision and speed with a model size of only 2.7 MB. This ensures high-performance foreign object detection on industrial conveyor belts without imposing additional hardware burdens. The comparative detection effects between the improved model and the original YOLOv8n are illustrated in Fig 14.

## 5 Conclusions and future work

1) This study proposes SCCG-YOLO, a lightweight detection model developed on the basis of YOLOv8n for large coal block detection on coal-mine conveyor belts. By integrating CPNGhost, ShuffleAttention, CARAFE, and the DIoU loss function, the proposed model improves feature extraction, background suppression, feature upsampling quality, and bounding-box regression accuracy under complex conveyor-belt scenarios. Experimental results show that SCCG-YOLO achieves 91.9% mAP@50 with only 2.7 MB parameters and an inference speed of 532.6 FPS, demonstrating a favorable balance among detection accuracy, model compactness, and real-time performance.
2) Ablation and comparative experiments further verify the effectiveness of the proposed improvements. The introduced modules contribute both independently and collaboratively to the final performance, and SCCG-YOLO shows competitive overall performance compared with several mainstream lightweight detectors. These results indicate that the proposed method has good potential for real-time warning applications in high-throughput conveyor-belt systems.
3) Nevertheless, the applicability of SCCG-YOLO still has clear boundaries. The current study is validated on a curated derivative subset of the public CUMT-Belt dataset, and the model is mainly oriented toward large-coal detection under similar imaging viewpoints, target definitions, and operating conditions. Therefore, its generalization capability under more diverse scenarios, such as severe illumination fluctuation, dense dust interference, motion blur, complex occlusion, different coal types, or cross-site domain shift, remains to be further verified. In addition, the current framework focuses on a single target category and has not yet been systematically evaluated for multi-category impurity detection tasks involving gangue, metal debris, tools, or mixed foreign materials. Hardware dependence is also a practical consideration, since the present experiments were conducted on a high-performance GPU platform, and the deployment effectiveness in resource-constrained embedded environments still requires dedicated verification.
4) Future work will therefore focus on three aspects. First, multimodal data fusion will be introduced to improve detection robustness under challenging mine conditions, for example by combining RGB images with infrared or depth-related information to enhance perception under low illumination, heavy dust, and partial occlusion. Second, embedded deployment verification will be carried out on mainstream edge devices such as Jetson Xavier NX and Orin Nano, with comprehensive evaluation of inference speed, computational resource occupancy, power consumption, and end-to-end deployment feasibility in practical scenarios. Third, the current single-category framework will be extended toward multi-category impurity detection, including gangue, metal debris, and other typical foreign objects on conveyor belts, so as to improve the extensibility and engineering value of the proposed method in intelligent mine monitoring systems.

## Supporting information

S1 File Formula derivation of CPNGhost module. (DOCX)

S2 File Formula derivation of SA module. (DOCX)

S3 File Formula derivation of CARAFE. (DOCX)

S4 File Formula derivation of DIoU. (DOCX)
