# Supplementary material for: Research on large coal detection method for mine conveyor belt based on SCCG-YOLO
Source: PLoS One. 2026 Apr 1;21(4):e0330980. doi: 10.1371/journal.pone.0330980 (PMC13042744; doi:10.1371/journal.pone.0330980)
Supplement: S1 File — (DOCX) [file pone.0330980.s001.docx]

Let the input feature be denoted as, the output after CPN convolution can be expressed as:

$$\mathbf{U}^{(0)}=\mathbf{X},\mathbf{U}^{(i)}=\text{SiLU}(\text{BN}_{i}(\mathbf{U}^{(i-1)}*\mathbf{W}_{i})),i=1,2,\ldots n$$

In the equation, * denotes the standard convolution operation, ***W****_i_* represents the convolution kernel parameters at the *i*-th layer, and *BN_i_* denotes the normalization operation at the *i*-th layer, with *n* typically set to 2.

The features processed by the CPN are then fed into the main convolution layer to generate the intrinsic feature map ***F***_int_:

$$\mathbf{F}_{\mathrm{int}}=\mathbf{U}^{_{(n)}}*\mathbf{W}_{1}$$

In the equation, ,*C*′ denotes the number of intermediate output channels.

The CPN module effectively expands the receptive field, enhancing the network’s capability to capture the global structure of large-sized objects. This allows the network to more comprehensively recognize the edges and contour shapes when processing images of large coal blocks.

Ghost Convolution, as a lightweight convolution operator, introduces the concept of feature decomposition. It first generates a subset of intrinsic features through primary convolution and then produces redundant features via inexpensive linear transformations. This approach expands the channel dimension of feature maps and enhances feature diversity without significantly increasing computational cost. The Ghost transformation is formulated as:

In the equation, *R*_j_ denotes the inexpensive transformation function, such as a 1×1 convolution or depthwise convolution, and *k* represents the number of redundant feature branches.

Finally, the CPN features and Ghost features are concatenated along the channel dimension to form the output feature map of the CPNGhost module:

The output channel number of this module is (*k*+1)*C*′, which significantly enhances feature diversity. This effectively alleviates the limitations of traditional convolution in feature representation when dealing with complex backgrounds or occluded objects.
