# Supplementary material for: Research on large coal detection method for mine conveyor belt based on SCCG-YOLO
Source: PLoS One. 2026 Apr 1;21(4):e0330980. doi: 10.1371/journal.pone.0330980 (PMC13042744; doi:10.1371/journal.pone.0330980)
Supplement: S2 File — (DOCX) [file pone.0330980.s002.docx]

The core innovation of the SA module lies in constructing a lightweight dual-attention enhancement mechanism through channel grouping, attention modeling, and channel shuffling operations. Given an input feature map, it is first divided along the channel dimension into two subsets,and, which are then fed into the channel attention branch and the spatial attention branch, respectively.

In the channel attention pathway, global average pooling is applied to extract the statistical features of each channel:

$$\mathbf{F}_{\text{avg}}^{\text{c}}=\frac{1}{H\times W}\sum_{i=1}^{H} \sum_{j=1}^{W} \mathbf{X}_{1} (C,i,j)$$

The pooled features are then passed through two fully connected layers, or equivalently, two 1×1 convolution layers, followed by a combination of activation functions to generate the channel attention vector :

$$\mathbf{M}_{\text{c}} =\sigma(W_{2} \cdot\delta(W_{1} \cdot\mathbf{F}_{\text{avg}}^{\text{c}} ))$$

In the equation, *δ* denotes the ReLU activation function, and *σ* represents the Sigmoid function. The resulting attention weights are then applied to the original channel features through channel-wise multiplication:

$$\mathbf{X}_{1}^{'}=\mathbf{M}_{\text{c}} \otimes\mathbf{X}_{1}$$

Meanwhile, in the spatial attention pathway, both max pooling and average pooling operations are performed along the channel dimension to obtain spatial information maps:

$$\mathbf{F}_{\text{max}}^{\text{s}}=\text{max}(\mathbf{X}_{2} ,\text{dim}=C)$$

$$\mathbf{F}_{\text{avg}}^{\text{s}}=\text{mean}(\mathbf{X}_{2} ,\text{dim}=C)$$

The two spatial descriptors are then concatenated and passed through a 7×7 convolution to generate the spatial attention map :

$$\mathbf{M}_{\text{s}} =\sigma(f^{7\times7}([\mathbf{F}_{\text{avg}}^{\text{s}} ;\mathbf{F}_{\text{max}}^{\text{s}}]))$$

The output is the weighted spatial feature map:

$$\mathbf{X}_{2}^{'}=\mathbf{M}_{\text{s}} \otimes\mathbf{X}_{2}$$

Subsequently, $\mathbf{X}_{1}^{'}$ and $\mathbf{X}_{2}^{'}$ are concatenated along the channel dimension, followed by a Shuffle operation—i.e., channel reordering—to enhance information fusion across the sub-channels, yielding the final output feature:
