# Supplementary material for: Research on large coal detection method for mine conveyor belt based on SCCG-YOLO
Source: PLoS One. 2026 Apr 1;21(4):e0330980. doi: 10.1371/journal.pone.0330980 (PMC13042744; doi:10.1371/journal.pone.0330980)
Supplement: S3 File — (DOCX) [file pone.0330980.s003.docx]

First, the kernel prediction module generates a set of reassembly kernels at each spatial location based on the input low-resolution feature map ​. Then, the content reassembly module applies these kernels to reweight and reorganize the original feature map, producing the upsampled high-resolution feature map ​, where *s* denotes the upsampling scale factor .

Compared to fixed interpolation methods, CARAFE adaptively allocates reassembly weights based on contextual information, enabling better preservation of object contours and texture details .

2.3.1. The kernel prediction module

For the input feature map ***X***, the kernel prediction module first applies a 1×1 convolution to map the channel dimension from *C* to *C*_mid_, thereby reducing computational complexity:

Subsequently, an *k*×*k* sliding window is applied to ***F*** through an unfold operation, extracting *k*^2^ feature vectors around each spatial location. A subsequent 1×1 convolution maps the channel dimension to *s*^2^×*k*^2^, thereby generating reassembly weights of size *k*^2^ for each upsampling position:

Next, a softmax function is applied along the channel dimension of the generated kernel weights to normalize them within each *k*^2^-sized window:

In the equation, denotes the *i* weight corresponding to the *j* position in the original feature map.

2.3.2. The content reassembly module

The core idea of the content reassembly module is to reweight and sum the *k*×*k* features within the receptive field of the input feature map for each output position, using the predicted reassembly weights. For the position (*u*,*v*) in the upsampled feature map Y, the corresponding center position in the input feature map is:$\left(\left\lfloor\frac{u}{s}\right\rfloor,\left\lfloor\frac{v}{s}\right\rfloor\right)$.The *k*×*k* neighborhood of feature vectors centered at this position can be represented as:

$$\{\mathbf{X}_{c,i+a,j+b} \text{∣}a,b\in\{0,\ldots,k-1\}\}$$

In the equation,$i=\left\lfloor\frac{u}{s}\right\rfloor,j=\left\lfloor\frac{v}{s}\right\rfloor$. The reassembly process can thus be expressed as:

Since is generated adaptively for each position, CARAFE is capable of assigning optimal reassembly weights according to different scenarios and object sizes . For example, when detecting the edges of large coal block impurities, the model can emphasize features near the object boundaries, whereas in flatter regions, it can prioritize broader contextual information.
