# Supplementary material for: Research on large coal detection method for mine conveyor belt based on SCCG-YOLO
Source: PLoS One. 2026 Apr 1;21(4):e0330980. doi: 10.1371/journal.pone.0330980 (PMC13042744; doi:10.1371/journal.pone.0330980)
Supplement: S4 File — (DOCX) [file pone.0330980.s004.docx]

DIoU is designed to comprehensively consider the overlap area between the predicted and ground truth boxes, the distance between their center points, and the bounding relationship of the boxes, thereby providing a more holistic measure of geometric differences. The DIoU loss is defined as:

$L_{\text{DIoU}} =1-\text{IoU}+\frac{\rho^{2}(b,b^{\text{gt}})}{c^{2}}$ (1)

In the equation, IoU denotes the intersection over union between the predicted and ground truth bounding boxes; ρ(b,bgt) represents the Euclidean distance between the center points of the predicted box bbb and the ground truth box bgt; and c is the diagonal length of the smallest enclosing box covering both bounding boxes. Compared with the original IoU loss, the DIoU loss introduces a center-point distance penalty term, which significantly improves the localization accuracy and convergence speed of the model.
